# Supplementary material for: Mapping of Metabolic Heterogeneity of Glioma Using MR-Spectroscopy
Source: Cancers (Basel). 2021 May 17;13(10):2417. doi: 10.3390/cancers13102417 (PMC8155922; doi:10.3390/cancers13102417)
Supplement: Supplementary file 1 [file cancers-13-02417-s001.zip › cancers-1165128-supplementary-final/Supplementary Tables.pdf]

Supplementary Table S1 - Clinical and demographic characteristics of the patient population included in this study

Characteristic	N=90	%
**Gender**		
Female	38	42.2
Male	53	58.9
**Age (years)**		
Median	52.6	
Range	(16-84)	
**Lesion type**		
Glioma	65	73
Metastasis	7	7.8
Ependymoma	1	1.1
Epidermoid	1	1.1
Meningioma	1	1.1
Pineal gland tumor	2	2.2
NHL	1	1.1
FCD	3	3.3
DNET	2	2.2
Ganglioglioma	1	1.1
Encephalomyelitis	2	2.2
Gliosis	2	2.2
ICH	1	1.1
Ischemia	1	1.1
**Localization**		
frontal	34	37.7
temporal	22	24.4

parietal	17	18.9
occipital	8	8.9
Pineal gland	2	2.2
posterior fossa	1	1.1
whole brain	2	2.2
insular	3	3.3
Cingular gyrus	1	1.1
Corpus callosum	1	1.1

Supplementary Table S2 – IDH status and histology from glioma patients included in this study

**Characteristic**	**N=65**	**(%)**
**IDH-Status**		
IDH-wildtype	35	53.8
IDH-mutated	17	26.2
IDH-mutated + LOH	13	20
**Histological Diagnosis**		
Diffuse Astrocytoma (WHO Grade II)	6	9.2
Anaplastic Astrocytoma (WHO Grade III)	11	16.9
Glioblastoma (WHO Grade IV)	35	53.8
Oligodendroglioma (WHO Grade II)	5	7.7
Anaplastic Oligodendroglioma (WHO Grade III)	8	12.3
