# Supplementary figures and images for: Mapping of Metabolic Heterogeneity of Glioma Using MR-Spectroscopy
Source: Cancers (Basel). 2021 May 17;13(10):2417. doi: 10.3390/cancers13102417 (PMC8155922; doi:10.3390/cancers13102417)

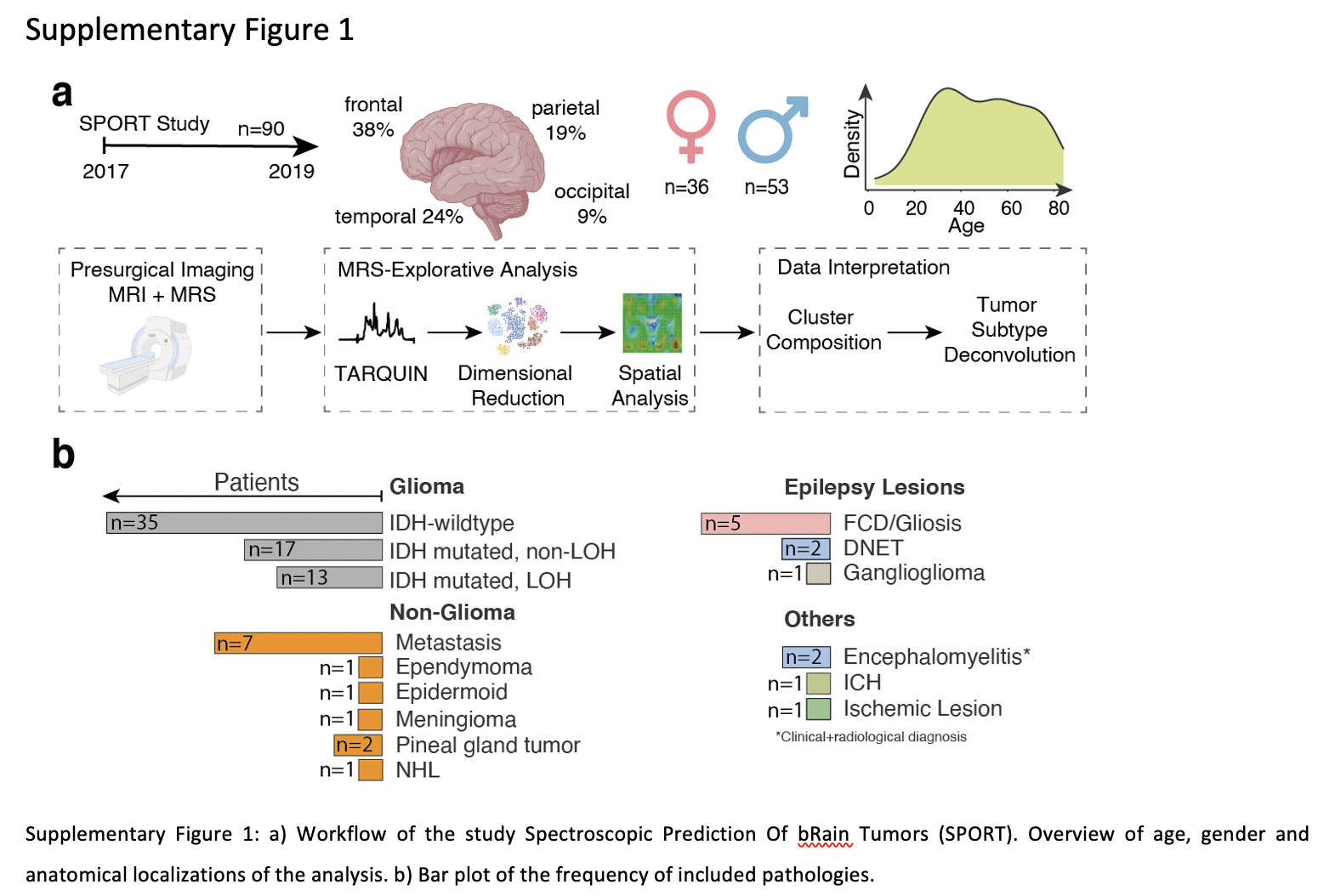

Supplement: Supplementary file 1 [file cancers-13-02417-s001.zip › cancers-1165128-supplementary-final/Figure S1.png]

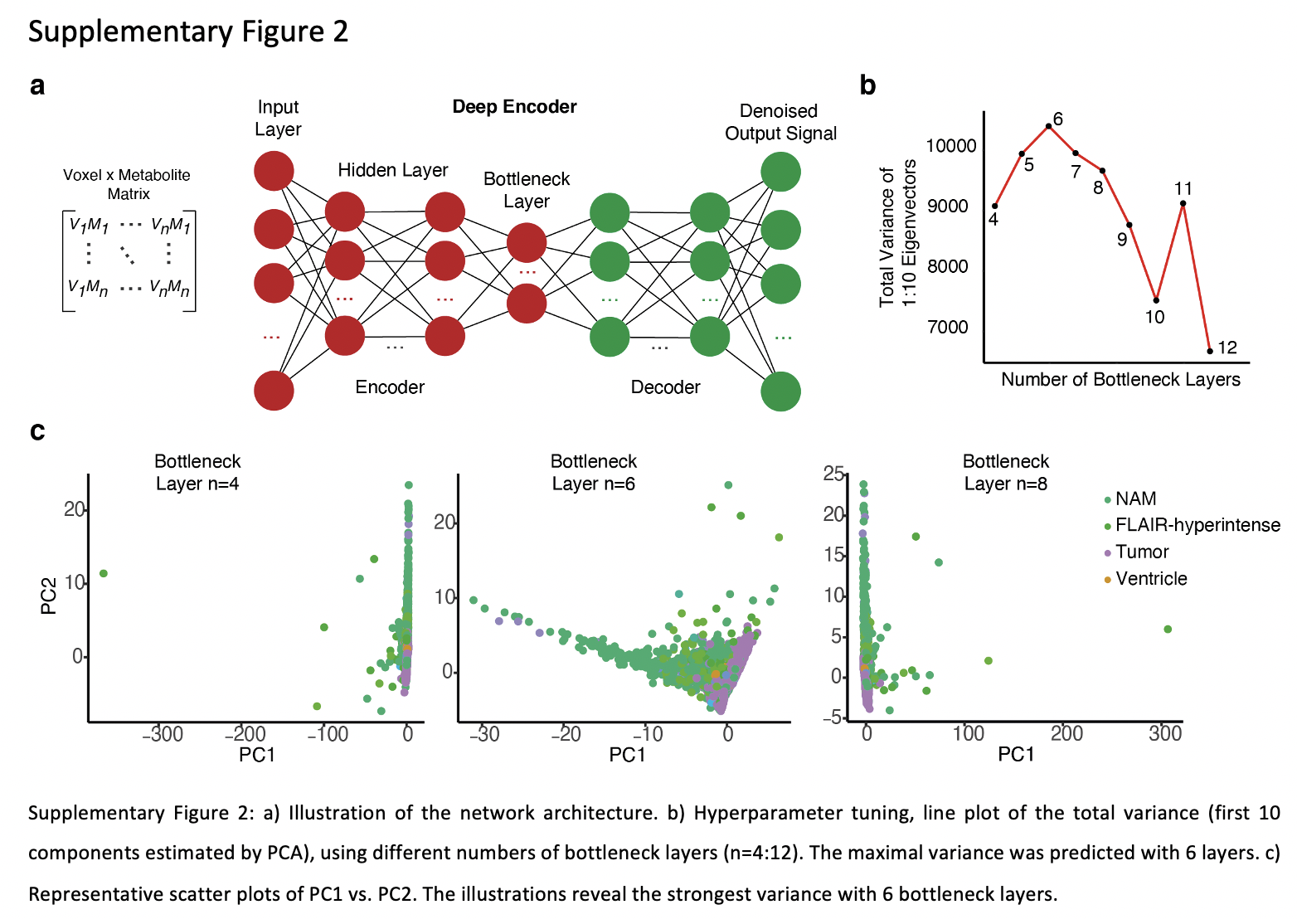

Supplement: Supplementary file 1 [file cancers-13-02417-s001.zip › cancers-1165128-supplementary-final/Figure S2.png]

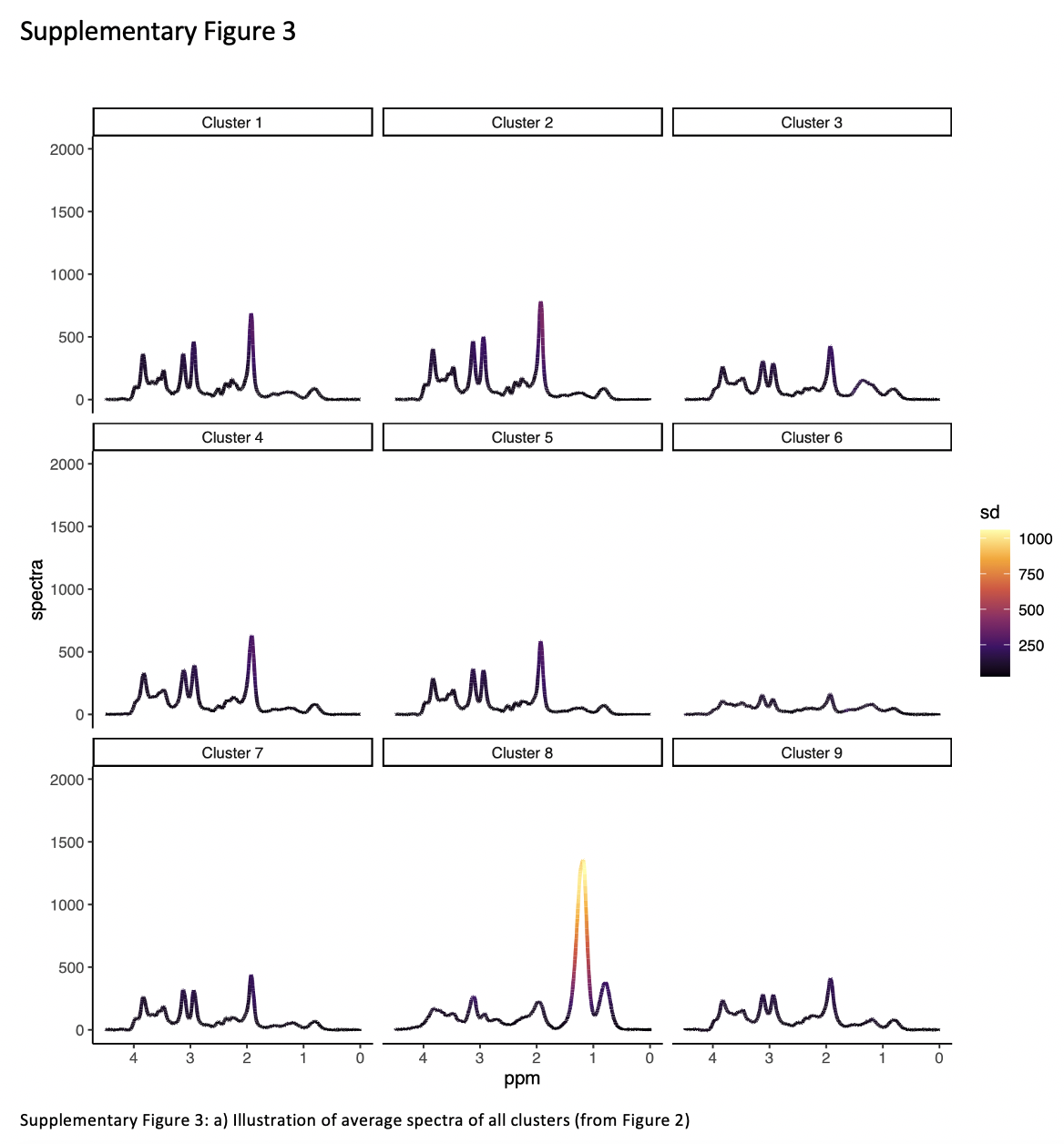

Supplement: Supplementary file 1 [file cancers-13-02417-s001.zip › cancers-1165128-supplementary-final/Figure S3.png]
